# Determination of the foraging behaviour and blood meal source of malaria vector mosquitoes in Trincomalee District of Sri Lanka using a multiplex real time polymerase chain reaction assay

**DOI:** 10.1186/s12936-016-1279-5

**Published:** 2016-04-26

**Authors:** Nayana Gunathilaka, Thanuja Denipitiya, Menaka Hapugoda, Wimaladharma Abeyewickreme, Rajitha Wickremasinghe

**Affiliations:** Department of Parasitology, Faculty of Medicine, University of Kelaniya, Ragama, Sri Lanka; Molecular Medicine Unit, Faculty of Medicine, University of Kelaniya, Ragama, Sri Lanka; Department of Public Health, Faculty of Medicine, University of Kelaniya, Ragama, Sri Lanka; Tropical & Environmental Diseases & Health Associates, No 3 Elibank Rd, Colombo 5, Sri Lanka

**Keywords:** *Anopheles*, Malaria, Mosquitoes

## Abstract

**Background:**

Studies of host preference patterns in blood-feeding anopheline mosquitoes are crucial to incriminating malaria vectors. However, little information is available on host preferences of *Anopheles* mosquitoes in Sri Lanka.

**Methods:**

Adult *Anopheles* mosquitoes were collected from five selected sentinel sites in Trincomalee District during June–September 2011. Each blood-fed mosquito was processed on filter papers. DNA was extracted using the dried blood meal protocol of the QIAmp DNA mini kit. A multiplexed, real-time PCR assay targeting eight animals was developed for two panels to identify the host meal of *Anopheles*. Human blood index (HBI), forage ratio (FR) and host feeding index (HFI) were calculated.

**Results:**

A total of 280 field-caught, freshly engorged female mosquitoes belonging to 12 anopheline species were analysed. The overall HBI and HFI in the present study were low indicating that humans were not the preferred host for the tested anopheline species. Nevertheless, a small proportion engorged *Anopheles aconitus*, *Anopheles culicifacies*, *Anopheles barbirostris*, *Anopheles annularis*, *Anopheles subpictus*, *Anopheles peditaeniatus*, *Anopheles pseudojamesi*, and *Anopheles barbumbrosus* contained human blood.

**Conclusion:**

The presence of human blood in mosquito species indicates the possibility of them transmitting malaria. Further studies on vector competence are needed to determine the role of each of the above anopheline species as efficient vectors of malaria.

## Background

The protracted history of malaria in Sri Lanka has been studied: some landmark events in global public health, such as the severe malaria epidemic of the 1930s, which led to an estimated 5.5 million cases and 80,000 reported deaths, and near-elimination of malaria during the Global Malaria Eradication Programme of the 1960s, which reduced the number of cases from 91,990 in 1953 to a mere 17 in 1963, many of these being imported infections [1].

In the past decade, the National Malaria Control Programme of Sri Lanka, the Anti-Malaria Campaign (AMC), has once again achieved a steady reduction in malaria transmission rates in the country. These have been sustained for more than 10 years over a period that spanned a 30-year separatist war in the north and east of the country, areas that were previously highly malarious [2]. There have now been no indigenous malaria cases in the country among its 20 million inhabitants since October 2012 [1, 3].

*Anopheles culicifacies* was regarded as the only malaria vector in the country until the early 1980s. In addition to *An. culicifacies,* enzyme-linked immunosorbent assay (ELISA)-based evidence has shown a large number of anopheline species to be infected with malaria parasites. These include *Anopheles aconitus*, *Anopheles annularis*, *Anopheles barbirostris*, *Anopheles nigerrimus*, *Anopheles pallidus*, *Anopheles subpictus*, *Anopheles tessellatus*, *Anopheles vagus*, and *Anopheles varuna*. Among these, species to have been consistently incriminated as malarial vectors are *An. annularis*, *An. subpictus*, *An. varuna*, and *An. tessellatus* [4, 5].

Anophelines exhibit a wide range of host preferences, such as humans, livestock, birds, and reptiles [6]. The vectorial capacity of a mosquito species for human pathogens is determined by several factors, such as mosquito population density, intrinsic incubation period of each pathogen, probability of daily survival of the vector, and host-seeking behaviour [7]. Man-biting behaviour is calculated from the human blood index (HBI) and the feeding frequency of mosquitoes. The HBI of mosquitoes, the proportion of blood meals taken from humans, is species-specific and affected by the availability of human hosts. Therefore, it is possible to hypothesize that differences in host preference contribute to apparently weak vectorial capacity of mosquito vectors in field conditions. It is thus important to understand the feeding behaviour of mosquito vectors as a prerequisite to determine its role in disease transmission in endemic settings. Host preference studies have also been used to monitor the effectiveness of vector control programmes by observing a reduction in blood-feeding behaviour, and have served as evidence of control failure [8–10].

However, little information is available regarding bionomic studies, such as host preferences and blood-feeding behaviour of *Anopheles* species in Sri Lanka. Methods for determining the blood meal source of hematophagous insects have evolved over the years. Precipitin tests have been used to detect the blood meal source of insects for many decades. In the 1980s an ELISA was introduced as a more sensitive alternative [11]. Both methods have their advantages and disadvantages and were often chosen based on the situation and level of accuracy, specificity and sensitivity required [12]. Other methods, such as haemoglobin crystallization [13], agglutination reactions [14] and immunofluorescence [15] have either been proposed or used. Although each method has proven useful, they are either inadequately sensitive, specific or time consuming [16, 17].

Laboratories that process large number of mosquito specimens for multiple diagnostic features require methods and processes that have high throughput, and are efficient and cost effective. The polymerase chain reaction (PCR)-based assay is more sensitive, less costly and less time consuming. The objective of this study was to determine the host preference of anopheline mosquitoes in Sri Lanka using a real-time PCR assay.

## Methods

### Study area

**Fig. 1:**
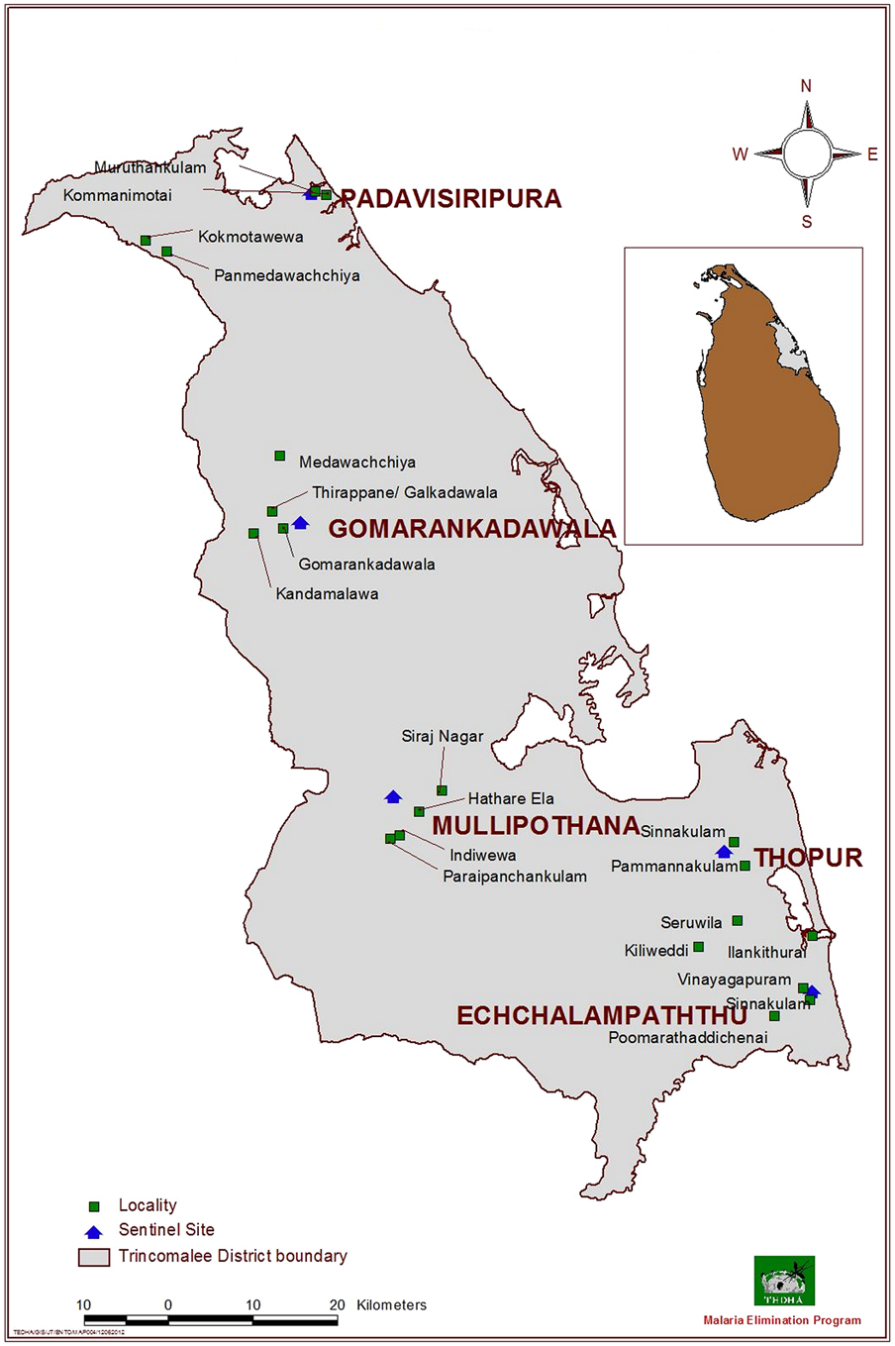
Map showing sentinel sites in the District of Trincomalee

Five sentinel sites: Gomarankadawala (E 0494617, N 0958609), Echchalampaththu (E 0540791, N 0918221), Mullipothana (E 0506055, N 0934096), Thoppur (E 0534419, N 0929257), and Padavisiripura (E 0498928, N 0988280) were selected in the Trincomalee District (Eastern Province), which is in the dry zone of Sri Lanka, for surveillance in consultation with the National Malaria Control Programme (Fig. 1). Factors such as past malaria history, availability of breeding sites, an established agricultural community, and feasibility of field operations to collect relevant data were considered in selecting the study areas.

The study areas had an average temperature ranging from 24.8 to 30.7 °C and an annual rainfall of 1649 mm during the study period [18, 19]. Mosquito breeding sites within the villages included a variety of habitats: slow-running streams, wells, pools, ponds, wastewater drains, and rice fields. The majority of houses in the study areas were made of cement with tin roofs and open eaves for aeration. Doors and windows were normally kept open until people went to bed.

### Experimental design

Mosquitoes were collected from June to September 2011 by indoors hand collection (HC) using standard mouth aspirators recommended by World Health Organization (WHO) [20]. Collections were made from 06.00 to 08.30 h by two trained persons spending a maximum of 10 min per house. Collected mosquitoes were transferred to entomological laboratories in the sentinel sites and mosquitoes were knocked down using 70 % chloroform. Blood-fed *Anopheles* mosquitoes were separated based on the abdominal appearance using a dissecting microscope; female anophelines species were identified to species level with the help of morphological taxonomic keys prepared for Sri Lankan anophelines [19–21].

### Mosquito processing

Engorged female mosquitoes were processed on 9-cm Whatman filter papers (according to WHO protocols) within 24 h after collection [20]. One filter paper only was used for one species of mosquito obtained from the same type of resting site. Each paper was labelled with a reference number, species, date, and time of collection. Processed samples were transported to the laboratory at the Molecular Medicine Unit, Faculty of Medicine, University of Kelaniya for identification of blood meal types.

### DNA extraction

Genomic DNA from blood-fed mosquitoes was extracted using the QIAamp DNA Mini Kit (Qiagen GmbH, Hilden, Germany) and the dried blood spot protocol described by the manufacturer was followed.

### PCR primers

Species-specific primers (Table 1) for the detection of bovine, cat, pig, monkey, chicken, human, dog, and rat were taken for the sequences available in the Genbank database [22–24]. All primers were purchased from Integrated DNA Technologies (IDT), San Diego, CA, USA.

**Table 1:** Species-specific primers used

Species	Accession no.	Position	Primer sequence F: forward 5′–3′ and R: reverse 5′–3′)	Size of amplified product (bp)
Bovine	J01394	8107/8127	F: GCCATATACTCTCCTTGGTGACA	271
		8377/8357	R: GTAGGCTTGGGAATAGTACGA	
Cat	NC001700	7413/7434	F: TTCTCAGGATATACCCTTGACA	180
		7571/7592	R: GAAAGAGCCCATTGAGGAAATC	
Pig	AF039170	93/115	F: GCCTAAATCTCCCCTCAATGGTA	212
		304/281	R: ATGAAAGAGGCAAATAGATTTTCG	
Monkey	AF312703	320/339	F: CCTCTTTCCTGCTGCTAATG	222
		522/541	R: TTTGATACTGGGATATGGCG	
Chicken	X52392	9062/9086	F: GGGACACCCTCCCCCTTAATGACA	266
		9327/9307	R: GGAGGGCTGGAAGAAGGAGTG	
Human	J01415	5967/5988	F: TTCGGCGCATGAGCTGGAGTCC	228
		6173/6194	R: TATGCGGGGAAACGCCATATCG	
Dog	U96639	5466/5487	F: GAACTAGGTCAGCCCGGTACTT	153
		5597/5618	R: CGGAGCACCAATTATTAACGGC	
Rat	NC001665	6022/6043	F: CGGCCACCCAGAAGTGTACATC	196
		6196/6217	R: GGCTCGGGTGTCTACATCTAGG	

### Development of a multiplex quantitative real-time PCR assay

Two multiplex, quantitative, real-time PCR assays targeting all animals were developed using a two-panel set of primers. The specificity of each primer was validated by real time PCR using DNA from the afore-mentioned (bovine, cat, pig, monkey, chicken, human, dog, and rat) vertebrate species as positive controls.

The DNA samples from each of those animals were used to develop and optimize assay conditions of two multiplex PCR assays with SYBR Green real-time PCR kit (Thermo Scientific, CA, USA) using different sets of primers. Assay conditions were optimized to obtain a specific fluorescence. All channels were scanned simultaneously for increases in fluorescence following the extension of each cycle using Swift Spectrum 48 fluorescence quantitative PCR detection systems (SPT-RT-48, Esco Health Care Pvt Ltd, Singapore). Each sample was tested for panel-one and panel-two animals simultaneously.

### Data analysis

Fit point method was used to analyse data. The PCR software plots an amplification curve from a plot of relative fluorescence (Δ *R* n) versus the cycle number. A threshold cycle (Ct) is determined for each sample at the point it crosses a baseline value, which was determined by the software.

Only the samples, which cross the baseline in the fluorescence graph, were taken as positive. Melting curve, which plots the derivative (dF/dT) versus the temperature (T) by the software for each sample was further analysed in order to validate and confirm the final product. The melting temperature of each targeted animal was determined using its own primer set.

### Validation of results

The lower the Ct value, the higher the amount of starting template material. Samples that did not cross the baseline value before 30 cycles were determined to have no identifiable blood from a particular animal. The cut-off was set as Ct 30, the last cycle completely devoid of background noise.

### Multiplex real-time PCR amplification

The PCR amplifications were done in 30 μl of a solution containing 1X SYBR Green-1, 0.01 μM of forward primer, reverse primer and template DNA. Thirty-five cycles of PCR programme (94 °C at 30 s, 62 (Panel 1) or 69 °C (Panel 2) at 30 s and 72 °C at 30 s) was terminated by incubating the product at 72 °C for 20 min, followed by melting cycles from 72 to 94 °C with plate reading at every 0.5 °C for 10 s. The samples were kept at 4 °C to terminate the real-time PCR reaction. Nuclease-free water was used as the no-template control and DNA isolated from each animal was used as positive control in each panel. All channels were scanned simultaneously for increases in fluorescence following the annealing stage of each cycle. Sensitivity of the PCR was observed as 55.9 × 10^6^ to 50.0 ng.

### Calculation of blood indices

The HBI, defined as the proportion of freshly engorged mosquitoes containing human blood, was calculated as described by Garrett-Jones [7]. Mixed (human + bovine) blood meals were added to the number of human and bovine blood meals when calculating the HBI, forage ratio (FR) and host feeding index (HFI) separately.

The FR, which quantifies vector selection of a particular vertebrate host rather than other available hosts, was estimated [25]. FRs were calculated as the per cent of females containing blood of a particular host, divided by the per cent of total available hosts [26]. A FR of 1.0 indicates neither a selective bias nor avoidance of a host animal; FRs significantly >1.0 indicate a selective bias, and values <1.0 indicate avoidance of a host in favour of other available hosts.

The HFI, defined as the observed proportion of feeds on one host with respect to another divided by the expected comparative proportion of feeds on these two hosts, was calculated using the formula [27] as given below:

$${\rm HFI}=\frac{ {\rm N}_{\rm x} / {\rm N}_{\rm y}}{{\rm A}_{\rm x} / {\rm A}_{\rm y}}$$

N_x_ and N_y_ are the mean numbers of blood meals taken from hosts x and y per study site, respectively, and A_x_ and A_y_ are the mean numbers of hosts x and y per study site, respectively. An index of 1.0 indicates equal feeding on the two hosts; values <1.0 and >1.0 indicate a decrease or increase, respectively, in feeding on the first host relative to the second. HFIs were calculated for each pair of hosts. One advantage of the HFI over the FR is that the HFI does not require a full animal census [28].

### Statistical analysis

The variations of the host preferences of each anopheline species were investigated by devising PERMANOVA+. The calculated FR of each species was subjected to a principal coordinates (PCO) analysis followed by non-metric multi-dimensional scaling (NMDS) to analyse the means of the FRs of different anopheline species. The species with similar host preferences were recognized. In addition, a cluster analysis (with respect to Euclidean distance) followed by analysis of similarities (ANOSIM) was utilized for the visual representation and comparison of the statistical significance in FR variations between clustered samples of anopheline species tested.

## Results

### *Anopheles* species analysed for determination of blood meal origin

Overall, 280 freshly engorged females mosquitoes belonging to 12 anopheline species: *An. culicifacies* (n = 20), *An. subpictus* (n = 30), *An. annularis* (n = 15), *An. nigerrimus* (n = 25), *An. aconitus* (n = 30), *An. vagus* (n = 26), *An. pallidus* (n = 29), *Anopheles peditaeniatus* (n = 26), *Anopheles pseudojamesi* (n = 25), *Anopheles karwari* (n = 1), *An. barbirostris* (n = 28), *Anopheles barbumbrosus* (n = 25) were analysed.

### Feeding behaviour of *Anopheles* mosquitoes

The host preference of anophelines observed in this study was 86.27 % *Bos taurus* (bovine), 11.62 % *Homo sapiens* (human), 1.06 % *Felis catus* (cat), and 0.35 % *Sus scrofa* (pig). Only 9.15 % was positive for both human and bovine blood. In addition, 4.9 % of the total samples tested were of unidentified origin. *Anopheles annularis* showed the highest range of host feeding behaviour which denoted the presence of human, bovine and cat blood. *Anopheles nigerrimus* was identified as positive for bovine and cat blood, *Anopheles pallidus* and *An. pseudojamesi* species were positive only for the bovine blood, while all other species were positive for both human and bovine blood. Mixed (human + bovine) blood meals were identified from *An. aconitus* (n = 12), *An. barbirostris* (n = 8), *An. peditaeniatus* (n = 2), *An. subpictus* (n = 2), *An. pseudojamesi* (n = 1) and *An. barbumbrosus* (n = 1). The vertebrate host species identified for each mosquito species is shown in Table 2.

**Table 2:** Vertebrate host species identified and human blood index (HBI) for each mosquito species

Mosquito species	Host detected	Human blood index (HBI)
*An. aconitus*	*Homo sapiens* (human) *Bos taurus* (bovine)	0.36
*An. annularis*	*Homo sapiens* *Bos taurus* *Felis catus* (cat)	0.07
*An. barbirostris*	*Homo sapiens* *Bos taurus*	0.27
*An. barbumbrosus*	*Homo sapiens* *Bos taurus*	0.04
*An. culicifacies*	*Homo sapiens* *Bos taurus*	0.15
*An. karwari*	*Homo sapiens* *Bos taurus*	0
*An. nigerrimus*	*Bos taurus* *Felis catus*	0
*An. pallidus*	*Bos taurus*	0
*An. peditaeniatus*	*Homo sapiens* *Bos taurus*	0.08
*An. pseudojamesi*	*Homo sapiens* *Bos taurus* *Sus scrofa* (pig)	0.04
*An. subpictus*	*Homo sapiens* *Bos taurus* *Felis catus*	0.166
*An. vagus*	*Bos taurus*	0

### HBI of anopheline mosquitoes

Of the 12 anophelines tested, *An. culicifacies*, *An. subpictus*, *An. annularis*, *An. aconitus*, *An. peditaeniatus*, *An. pseudojamesi*, *An. barbirostris*, and *An. barbumbrosus* were detected with human blood meals. The highest HBI was observed for *An. aconitus* followed by *An. barbumbrosus*, *An. subpictus* and *An. culicifacies*. Human blood was not detected in *An. nigerrimus*, *An. vagus* and *An. pallidus*. The HBI calculated for each mosquito species is given in Table 2.

### FR of anopheline mosquitoes

The number of humans exceeded domestic animals resident in each house of this survey. The FR for humans was <1.0 for most of the anophelines, except *An. aconitus* (Table 3). The ratios calculated for other animals were >1.0, especially for cattle.

**Table 3:** Forage ratios (FR) of *Anopheles* mosquitoes

Reference number	Mosquito species	Forage ratio (FR)					
		Human	Bovine	Cat	Dog	Pig	Chicken
1	*An. aconitus*	1.01	18.67	0	0	0	0
2	*An. annularis*	0.13	6.05	0.78	0	0	0
3	*An. barbirostris*	0.53	15.72	0	0	0	0
4	*An. barbumbrosus*	0.12	55.00	0	0	0	0
5	*An. culicifacies*	0.46	23.80	0	0	0	0
6	*An. karwari*	0	16.84	0	0	0	0
7	*An. nigerrimus*	0.00	47.94	2.46	0	0	0
8	*An. pallidus*	0.00	49.94	0	0	0	0
9	*An. peditaeniatus*	0.13	16.56	0	0	0	0
10	*An. pseudojamesi*	0.07	4.22	0	0	6.68	0
11	*An. subpictus*	0.40	14.11	1.62	0	0	0
12	*An. vagus*	0.00	38.41	0	0	0	0

### HFI of anopheline mosquitoes

Calculation of the HFI for each pair of vertebrate hosts revealed that humans were the least preferred source of blood relative to bovine, cat, dog, and pig (Table 4). In congruence with the FR values, HFI data suggest that cattle are the preferred source of blood for all anophelines tested. However, small proportion of engorged females of *An. aconitus* (36 %), *An. barbirostris* (26.6 %), *An. subpictus* (16.6 %), *An. culicifacies* (15 %), *An. peditaeniatus* (8 %), *An. annularis* (7 %) *An. pseudojamesi* (4 %) and *An. barbumbrosus* (4 %) contained human blood.

**Table 4:** Host feeding index (HFI) for each pair of vertebrate hosts fed upon by *Anopheles*

Host 1 (X)	Host 2 (Y)	*An. aconitus*	*An. annularis*	*An. barbirostris*	*An. barbumbrosus*	*An. culicifacies*	*An. karwari*	*An. nigerrimus*	*An. pallidus*	*An. peditaeniatus*	*An. pseudojamesi*	*An. subpictus*	*An. vagus*
Human	Bovine	0.03	0.02	0.03	0.00	0.02	0.00	0.00	0.00	0.01	0.00	0.03	0.00
	Cat	0.80	0.16	0.44	0.11	0.34	0.00	0.00	0.00	0.20	0.11	0.25	0.00
	Pig	0.06	0.01	0.37	0.03	0.08	0.00	0.00	0.00	0.07	0.01	0.01	0.00
Bovine	Human	18.51	47.30	29.94	285.9	64.81	8.56	301.5	364.3	1.00	58.68	35.54	251.2
	Cat	27.63	7.74	13.07	32.81	22.31	0.47	1.85	23.56	2.24	6.32	13.94	16.25
	Pig	1.06	0.28	11.20	7.81	5.31	0.40	13.50	16.31	0.75	0.66	0.87	11.25
Cat	Human	0.00	6.11	0.00	0.00	0.00	0.00	0.00	0.00	0.00	0.00	1.32	0.00
	Bovine	0.00	0.11	0.00	0.00	0.00	0.00	0.00	0.00	0.00	0.00	0.00	0.00
	Pig	0.00	0.04	0.00	0.00	0.00	0.00	0.00	0.00	0.00	0.00	0.00	0.00
Pig	Human	0.00	0.00	0.00	0.00	0.00	0.00	0.00	0.00	0.00	89.20	0.00	0.00
	Bovine	0.00	0.00	0.00	0.00	0.00	0.00	0.00	0.00	0.00	1.58	0.00	0.00
	Cat	0.00	0.00	0.00	0.00	0.00	0.00	0.00	0.00	0.00	9.60	0.00	0.00

### Statistical analysis

**Fig. 2:**
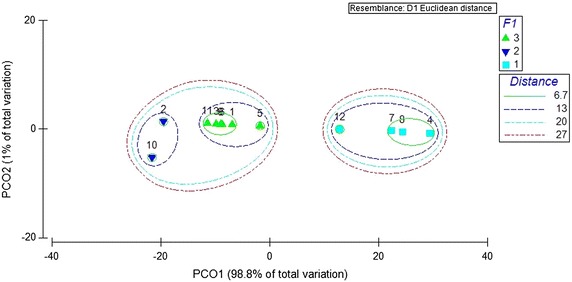
Principal coordinates (PCO) analysis of the forage ratio (FR) of anopheline mosquitoes based on PCO_1_ and PCO_2_ scores

A high cumulative percentage of 99.8 % of the total variation of blood meal was accounted by both PC_1_ and PC_2_ axis of the PCO, which suggested the presence of two major clusters of studied *Anopheles* mosquito species (12) at a Euclidean of distance of 27, in terms of their FRs. Furthermore, three sub-categories under two major clusters were noted at a Euclidean distance of 13 (Fig. 2).

*Anopheles annularis* and *An. pseudojamesi* composed one sub-category, while *An. aconitus, An. barbirostris, An. culicifacies, An. karwari, An. peditaeniatus,* and *An. subpictus* composed another sub-category. All the other species (*An. barbumbrosus*, *An. nigerrimus, An. pallidus*, and *An. vagus*) were observed as the third sub-category, in terms of the host preferences as indicated by FRs.

**Fig. 3:**
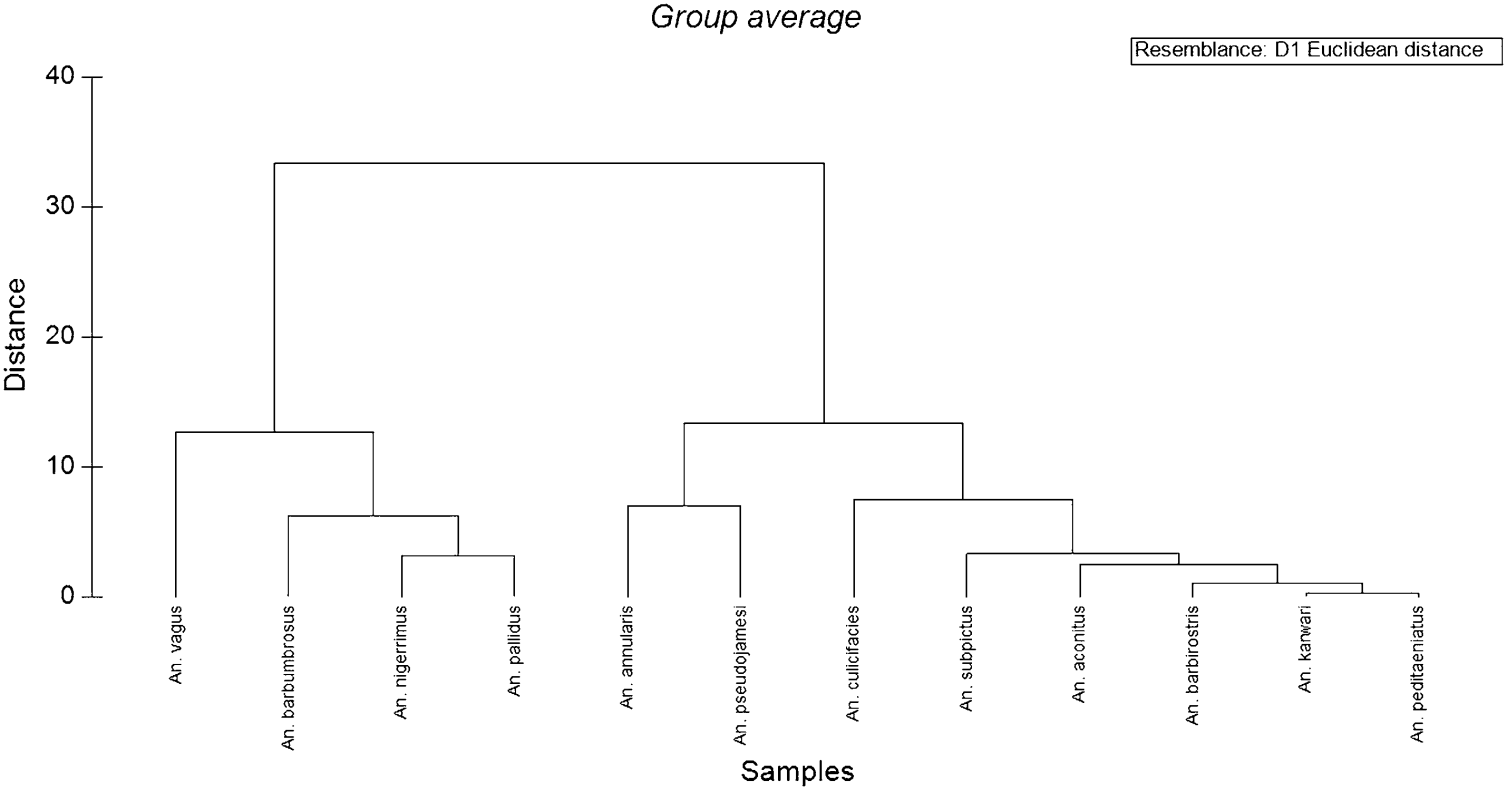
Dendrogram showing the clustering of anopheline mosquitoes based on the forage ratios (FRs)

**Fig. 4:**
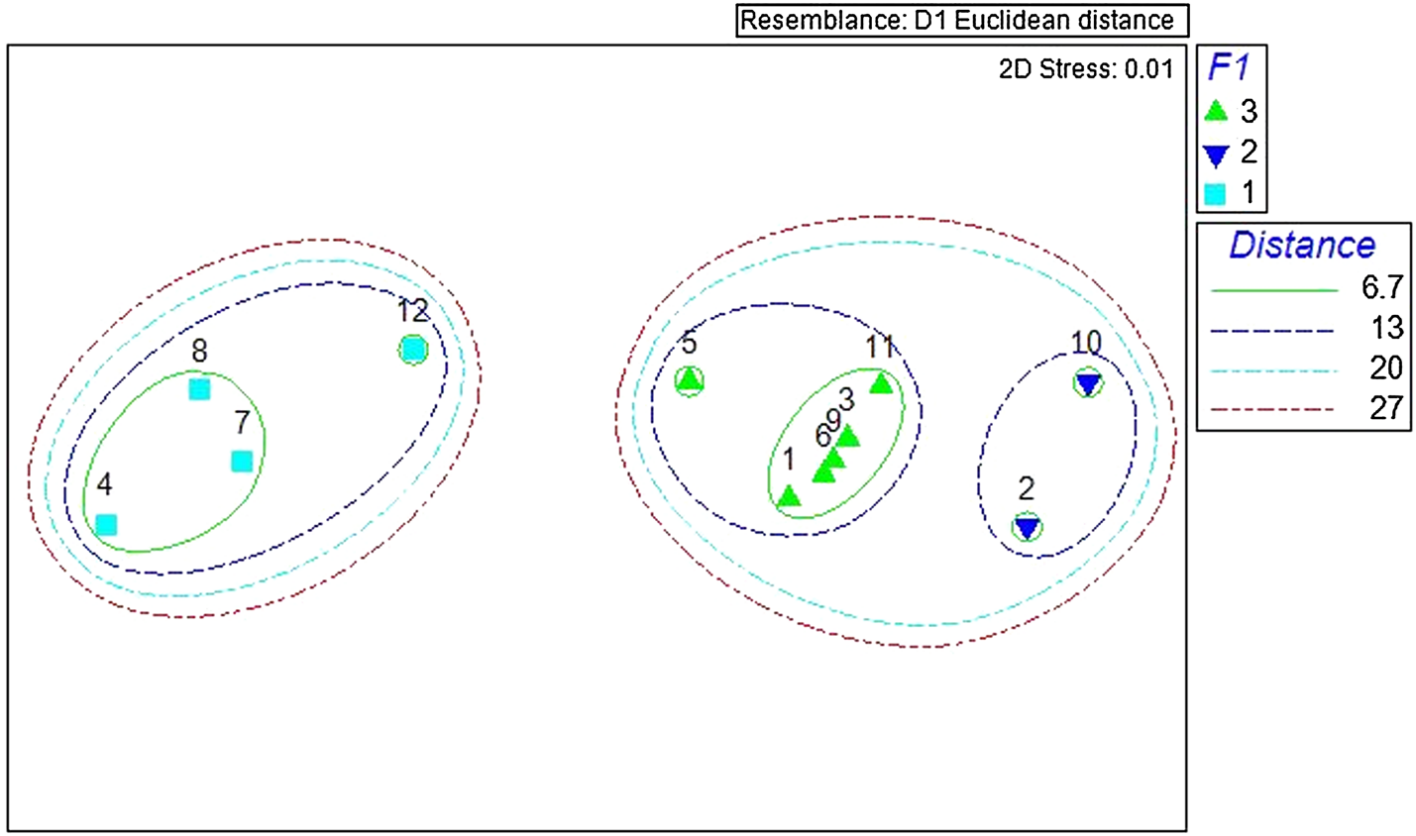
Non-metric multi-dimensional scaling (NMDS) analysis of mean value of the forage ratios (FRs) of different anopheline species

The NMDS which resulted from the MDS plot with satisfactory 2D stress value (0.01), demonstrated the emergence of the same three sub-categories, ensuring the reliability of the results obtained by the PCO (Fig. 3). The results of the ANOSIM (P < 0.05, Global R 0.944) and the dendrogram of the cluster analysis (Fig. 4, based on Euclidean distance) also confirmed the emergence of three clusters as statistically significant, confirming statistical difference on the host seeking behaviour of the anopheline species in this study.

## Discussion

Many epidemiologically important issues revolve around the question of host preference and feeding behaviour of vector mosquitoes. The incidence of malaria is influenced by mosquito host selection [5]. If mosquitoes do not discriminate between host species, the proportion of blood meals attributable to specific hosts would reflect the relative abundance of host animals.

Alternatively, certain mammalian species might be more attracted or accessible to specific mosquito species. Such host preferences, especially the degree of anthropophily, would affect the efficiency of malaria vectors. Climatic, environmental and socio-economic factors also influence vector populations by determining feeding behaviour and the vectorial capacity for malaria transmission [29]. Therefore, understanding the blood foraging pattern of insects in the field is important when implicating vectors [30]. However, little information is available regarding host preferences and blood-feeding behaviour of *Anopheles* species in Sri Lanka. This study was conducted to document the hematophagic tendencies of confirmed and suspected malaria vectors in malaria-endemic areas of Sri Lanka.

A census of humans and domestic animals at each selected household (n = 250) was taken by interviewing the chief occupant of each residence. Six vertebrate animals namely: bovine (n = 290), cat (n = 111), pig (n = 46) chicken (n = 908), dog (n = 147), and human (n = 1399) were identified. The number and composition of vertebrates remained same during the study period. However, due to practical constraints, the number of free ranging animals, temporarily and spatially associated with each study site, was not estimated.

The host preference of tested *Anopheles* was bovine, human, cat, and pig. Only 9.15 % were positive for both human and bovine. The 4.9 % of specimens from which blood meal source could not be identified, even after two repeat attempts, suggests that they may have taken blood meals from other domestic animals, peridomestic animals or any other wild animal, Based on the field observations, the origin of these blood meal sources would be peafowl (*Pavo* spp.), giant squirrel (*Ratufa macroura*), palm squirrel (*Funambulus palmarum*), gecko (*Hemidactylus* spp.), land monitor (*Varanus bengalensis*), ruddy mongoose (*Herpestes smithii*), elephant (*Elephas maximus*), spotted deer (*Axis axis*), porcupine (*Hystrix indica*), and black-naped hare (*Lepus nigricollis*). Other factors such as DNA degradation, enzyme inhibition and human error cannot be ruled out.

In this study, *An. subpictus, An. annularis, An. nigerrimus,* and *An. pseudojamesi* fed on three hosts. These results suggest that the local populations of these species may be opportunistic feeders. Studies on feeding behaviour of *An. culicifacies* show that it is predominantly zoophilic, feeding mainly on cattle (anthropophilic index less than 10 %), but where cattle are scarce its anthropophilic index can reach in excess of 20 % [31]. Previous studies conducted in the low country of Sri Lanka reported that among *An*. *culicifacies* and *An. subpictus* engorged females examined, 34.4 and 30.4 %, respectively, were multiple fed. In these two species, double meals accounted for 92.7 and 89.5 %, and triple meals for 7.3 and 10.5 % of multi-fed mosquitoes, respectively [32]. Studies on blood meals using ELISA showed that 8.3 % of *An. culicifacies* were human-fed and 80 % of these (i.e., 6.6 %) were concurrently bovid-fed [32].

This study revealed that among *An. culicifacies* no multiple blood meals were identified. About 15 % were human-fed and 85 % of these were bovid-fed. This indicates that *An. culicifacies*, which is considered as the major vector of malaria in Sri Lanka, may not be an opportunistic feeder on humans. It was also noted that *An. culicifacies* positive for either human or bovine blood. However, none of them were of mixed blood origin.

Among *An. subpictus* the majority (81.8 %) was bovid-fed and 6.6 % human-fed; 3.0 % were fed with cat blood and 3.0 % of fed mosquitoes were unidentified. About 6.1 % were positive for both human and bovine blood. Multiple blood feeding within the same gonotrophic cycle is attributed to a local ‘frequent feeding strategy’ in primarily zoophagic and endophilic malaria vectors [32]. About 6.6 % of *An. annularis* mosquitoes, considered a secondary vector of malaria in Sri Lanka, were fed with human blood; 86.6 % of them were positive for the blood of bovine and cat.

However, obtaining different blood meal sources by the same species during its life time may transmit the disease causative agents between different species and increase the danger of humans getting the diseases from animal origin. Therefore, blood meal analysis is essential to determine vector host choice and feeding behaviour changes, highlighting their importance in evaluating malaria control interventions and their incorporation into routine malaria control programmes.

The HBI values of all anopheline species were indicative of low anthropophily (lower than 0.5). It is possible that there are areas where the climate is optimal for malaria transmission and *Anopheles* mosquitoes, but there is no malaria. This may be due to the presence of *Anopheles* mosquitoes that do not primarily feed on humans [33]. Nevertheless, a small proportion engorged *An. aconitus*, *An. barbirostris*, *An. subpictus*, *An. culicifacies*, *An. peditaeniatus*, *An. annularis*, *An. pseudojamesi*, and *An. barbumbrosus* contained human blood. The FRs for human were <1.0 for most of the anophelines except *An. aconitus*.

Based on the HFIs for each pair of vertebrate hosts, humans were the least preferred blood source. In this study HFI could not be calculated for monkeys and rats as the wild population could not be enumerated. HFIs could not be calculated for dog and rat since those two animals were not detected from the samples. Based on these results, cattle were the preferred host for a blood meal by all *Anopheles* species tested.

These results suggest that zooprophylaxis and increased surveillance may be considered effective methods of preventing malaria transmission in malarious areas of Sri Lanka and the re-introduction of malaria in areas that have not reported malaria cases for a considerable period of time in achieving the goal of malaria elimination from Sri Lanka.

Laboratories that process large number of mosquito specimens for multiple diagnostic features require methods and processes that have high throughput, and are efficient and cost effective. Although the ELISA method is sensitive in terms of identifying the source of blood meals [34], it is time consuming, comparatively expensive and is limited in terms of throughput. The optimized multiplex PCR protocol for blood meal identification is more sensitive, less costly and less time consuming. It also provides a quantitative result rather than qualitative one.

The multiplex, real-time PCR assay, compared to other methods, appears to be quicker and less costly when assaying several blood meal sources. However, if the aim of identifying blood meal source is only to determine the proportion feeding on humans, such as determination of HBI, then ELISA and classical PCR is better in terms of cost and time.

A major advantage of the multiplex, real-time PCR is that it can be integrated with other molecular diagnostic methods, especially in laboratories that conduct multiple diagnostics on single specimens using extracted DNA of each specimen. Under these circumstances, apart from tailored primers, all the reagents used for blood meal identification by PCR are universal for any diagnostic PCR assay. Purchasing these reagents in large quantities can further reduce the cost for the PCR diagnostic process employed.

## Conclusion

The presence of human blood in mosquito species indicates the possibility of malaria transmission. Further studies on vector competence are needed to determine the role of the anopheline species that are currently efficient vectors of malaria. The optimized protocol can be used to determine the hematophagic tendencies of insect vectors.

## References

[1] Premaratne R, Ortega L, Janakan N, Mendis KN (2014). Malaria elimination in Sri Lanka: what it would take to reach the goal. South East Asian J Public Health.

[2] Abeyasinghe RR, Galappaththy GNL, Gueye CS, Kahn JG, Feachem RGA (2012). Malaria control and elimination in Sri Lanka: documenting progress and success factors in a conflict setting. PLoS One.

[3] Gunathilaka N, Abeyewickreme W, Hapugoda M, Wickremasinhe R (2015). Species composition, breeding habitat diversity and habitat characterization of malaria vector breeding habitats in Trincomalee District of Sri Lanka. Biomed Res Int.

[4] Amarasinghe PH, Amarasinghe FP, Kondadsen F, Fonseka KP, Wirtz RA (2001). Malaria vectors in a traditional dry zone village in Sri Lanka. Amer J Trop Med Hyg.

[5] Gunathilaka N, Hapugoda M, Abeyewickreme W, Wickremasinghe R (2015). Entomological investigations on malaria vectors in some war-torn areas in the Trincomalee District of Sri Lanka after settlement of 30-year of civil disturbance. Malar Res Treat.

[6] Subbarao SK (1998). Anopheline species complexes in South-East Asia.

[7] Garrett-Jones C (1964). The human blood index of malaria vectors in relation to epidemiological assessment. Bull World Health Organ.

[8] Lindsay SW, Snow RW, Broomfield GL, Janneh MS, Wirtz RA, Greenwood BM (1989). Impact of permethrin-treated bednets on malaria transmission by the *Anopheles gambiae* complex in The Gambia. Med Vet Entomol.

[9] Mathenge EM, Gimnig JE, Kolczak M, Ombok M, Irungu LW, Hawley WA (2001). Effect of permethrin-impregnated nets on exiting behavior, blood feeding success, and time of feeding of malaria mosquitoes (Diptera: Culicidae) in western Kenya. J Med Entomol.

[10] N’Guessan R, Corbel V, Akogbeto M, Rowland M (2007). Reduced efficacy of insecticide-treated nets and indoor residual spraying for malaria control in pyrethroid resistance area, Benin. Emerg Infect Dis.

[11] Burkot TR, Goodman WG, Foliart GR (1981). Identification of mosquito blood meals by enzyme-linked immunosorbent assay. Am J Trop Med Hyg.

[12] Gomes LAM, Duarte R, Lima DC, Diniz BS, Serrao ML, Labarthe N (2001). Comparison between precipitin and ELISA tests in the blood meal detection of *Aedes aegypti* (Linnaeus) and *Aedes fluviatilis* (Lutz) mosquitoes experimentally fed on feline, canine, and human hosts. Mem Inst Oswaldo Cruz.

[13] Washino RK, Else JG (1972). Identification of blood meals of hematophagous arthropods by the haemoglobin crystallization method. Am J Trop Med Hyg.

[14] Boorman J, Mellor PS, Boreham PFL, Hewett RS (1977). A latex agglutination test for the identification of blood meal of *Culicoides* (Diptera: Ceratopogonidae). Bull Entomol Res.

[15] McKinney RM, Spillane JT, Holden P (1972). Mosquito blood meals: identification by fluorescent antibody method. Am J Trop Med Hyg.

[16] Burkot TR (1988). Non-random host selection by anopheline mosquitoes. Parasitol Today.

[17] Osae M, Vezenegho S, Spillings B, Koekemoer L (2013). Optimization and validation of a multiplex PCR for identification of mammalian blood meals in malaria vector mosquitoes and time-cost comparison between the PCR and ELISA methods. Communi Dis Surveil Bull.

[18] Gunathilaka N, Fernando T, Hapugoda M, Wickremasinghe R, Wijeyerathne P, Abeyewickreme W (2013). *Anopheles culicifacies* breeding in polluted water bodies in Trincomalee district of Sri Lanka. Malar J.

[19] Gunathilaka N. Distribution of major and potential malaria vectors in Mannr and Trincomalee districts and systematics of anophelines in Sri Lanka. Sri Lanka: Ph.D thesis, University of Kelaniya. 2014.

[20] WHO (1992). Entomological field techniques for malaria control. Part I, Learners guide.

[21] Amerasinghe FP (1990). A guide to the identification of anopheline mosquitoes (Diptera: Culicidae) of Sri Lanka 1 adult females. Ceylon J Sci.

[22] Lahiff S, Glennon M, O’Brien L, Lyng J, Smith T, Maher M, Shilton N (2001). Species-specific PCR for the identification of ovine, porcine and chicken species in meat and bone meal (MBM). Mol Cell Probes.

[23] Parodi B, Aresu O, Bini D, Lorenzini R, Schena F, Visconti P (2002). Species identification and confirmation of human and animal cell lines: a PCR-based. Biotechniques.

[24] Chang MC, Teng HJ, Chen CF, Chen YC, Jeng CR (2008). The resting sites and blood-meal sources of *Anopheles minimus* in Taiwan. Malar J.

[25] Boreham PFL, Garrett JC (1973). Prevalence of mixed blood meals and double feeding in a malaria vector (*Anopheles sacharovi* Favre). Bull World Health Organ.

[26] Hess AD, Hayes RO, Tempelis C (1968). Use of forage ratio technique in mosquito host preference studies. Mosquito News.

[27] Richards SL, Ponnusamy L, Unnasch TR, Hassan HK, Apperson CS (2006). Host- feeding patterns of *Aedes albopictus* (Diptera: Culicidae) in relation to availability of human and domestic animals in suburban landscapes of central North Carolina. J Med Entomol.

[28] Kay BH, Boreham PFL, Williams GM (1979). Host preferences and feeding patterns of mosquitoes (Diptera: Culicidae) at Kowanyama, Cape York Peninsula, Northern Queensland. Bull Entomol Res.

[29] Mouchet J, Manguin S, Sircoulon J, Laventure S, Faye O, Onapa AW, Carnebale P (1998). Evolution of malaria in Africa for the past 40 years: impact of climate and human factors. J Am Mosq Control Assoc.

[30] Fujito S, Buei K, Nakajima S, Ito S, Yoshida M, Sonada H, Nakamura H (1971). Effect of the population density of *Culex tritaeniorhynchus* (Giles) on blood sucking rates in cowsheds and pigpens in relation to its role in the epidemic of Japanese encephalitis. Jpn J Sanit Zool.

[31] Vatandoost H, Emami SN, Oshaghi MA, Abai MR, Raeisi A, Piazzak N (2011). Ecology of malaria vector *Anopheles culicifacies* in a malarious area of Sistan va Baluchestan Province, South-East Islamic Republic of Iran. East Mediterr Health J.

[32] Amerasinghe PH, Amerasinghe FP (1999). Multiple host feeding in field populations of *Anopheles culicifacies* and *An. subpictus* in Sri Lanka. J Med Vet Entomol.

[33] Bruce-Chwatt LJ (1984). Essential Malariology.

[34] Burkot TR, Goodman WG, Foliart GR (1981). Identification of mosquito blood- meals by enzyme-linked immunosorbent assay. J Trop Med Hyg.

